# Ventilation/Perfusion SPECT for Diagnosis of Pulmonary Embolism and Other Diseases

**DOI:** 10.1155/2011/682949

**Published:** 2010-12-19

**Authors:** Marika Bajc, Björn Jonson

**Affiliations:** Department of Clinical Physiology, Lund University, 22185 Lund, Sweden

## Abstract

V/P_SPECT_ has the potential to become a first hand tool for diagnosis of pulmonary embolism based on standardized technology and new holistic interpretation criteria. Pretest probability helps clinicians choose the most appropriate objective test for diagnosis or exclusion of PE. Interpretation should also take into account all ventilation and perfusion patterns allowing diagnosis of other cardiopulmonary diseases than PE. In such contexts, V/P_SPECT_ has excellent sensitivity and specificity. Nondiagnostic reports are ≤3%. V/P_SPECT_ has no contraindication; it is noninvasive and has very low radiation exposure. Moreover, acquisition time for V/P_SPECT_ is only 20 minutes. It allows quantification of PE extension which has an impact on individual treatment. It is uniquely useful for followup and research.

## 1. Introduction

Prior to the development of CT angiography, planar ventilation/perfusion scans were the primary noninvasive method for diagnosis of pulmonary embolism (PE). However, the technique suffered disrepute since the PIOPED I study showed that 65% of scans were nondiagnostic [1]. As will be reviewed below, results from later studies based upon modern imaging techniques and new holistic principles (combining clinical information, pretest probability, results of chest radiograph, and patterns typical of PE or other diseases) reduce the number of nondiagnostic findings to 3% or less, while sensitivity and specificity are excellent [2, 3]. 

Since the early 1980s, the advantage of tomography over planar imaging for PE detection was indicated [4]. Since then, numerous studies have shown such advantages of ventilation/perfusion single photon emission computed tomography (V/P_SPECT_) over alternative techniques, which indicated that lung scintigraphy is again appreciated as a first line method for diagnosis of PE.

An important issue is to estimate the clinical probability for PE before performing imaging tests as is elaborated upon in the European Guidelines for Lung Scintigraphy [2] and by Mamlouk et al. [5]. The object of this paper is to show the advantages of V/P_SPECT_ in accordance with the European Guidelines for V/P_SPECT_ [2, 3]. It will be emphasized that V/P_SPECT_ gives diagnostic information in other conditions such as pneumonia, COPD, and left heart failure. The presentation will focus on basic requirements on diagnostic methods for PE:

1. fast procedure,
2. low radiation dose,
3. no contraindications,
4. high diagnostic accuracy and few nondiagnostic reports,
5. utility for selection of treatment strategy,
6. suitability for followup and research.

## 2. Agents Used for Imaging of Ventilation

Gases are distributed strictly according to regional ventilation. The only gas that is useful for V/P_SPECT_ is krypton, ^81m^Kr. Its short half life (13 s) implies that it disappears from the alveoli by decay rather than by exhalation. After some minutes of test gas breathing, when the alveolar concentration has approached a steady state reflecting alveolar ventilation, V/P_SPECT_ is performed. The rubidium generator that delivers ^81m^Kr has a half life of 4.6 h. Limited availability and high costs prevent a general use of ^81m^Kr.

Inhalation of a radio-aerosol is used in nearly all centers for ventilation scintigraphy. Aerosol particles are liquid or solid. Particles larger than 2 *μ*m are deposited in large airways (hot spots). Smaller particles are deposited by sedimentation and diffusion in small airways and alveoli. Particles smaller than 1 *μ*m are mainly deposited in alveoli by diffusion. Aerosol deposition is modified by flow pattern. High flow rates at forced breathing patterns and turbulent flow enhance particle deposition in airways and augment tendencies to hot spots in ventilation images, particularly in Chronic Obstructive Pulmonary Disease (COPD). 

The mass median aerodynamic diameter, MMAD, reflects radioactivity carried by each liquid particle. Half of the radioactivity resides in particles smaller than MMAD and 50% in larger ones. It is often recommended that the maximum droplet size inhaled by the patient should not exceed 2 *μ*m. Because of the complex physics behind aerosol deposition pattern, the performance of a nebuliser must be clinically tested.

Diethylenetriaminepentaacetic acid labelled with technetium, ^99m^Tc-DTPA, is in general use for ventilation scintigraphy with liquid aerosols. The size of the water solvable molecule is 492 Dalton [6]. Therefore, ^99m^Tc-DTPA diffuses through the alveolocapillary membrane to the blood. In a healthy patient, clearance of ^99m^Tc-DTPA occurs with a half life of about 70 minutes. Increased clearance, leading to a shorter half life is observed with alveolar inflammation of any kind, such as alveolitis of allergic or toxic nature and even in smokers [7–9]. 

Technegas is an aerosol of extremely small carbon particles, 0.005–0.2 *μ*m, generated in a high temperature furnace [10–12]. The small particle size implies that they are distributed in the lungs almost like a gas and are deposited in alveoli by diffusion [13, 14]. Technegas provides images which are equivalent to those with ^81m^Kr [14–18]. 

**Figure 1:**
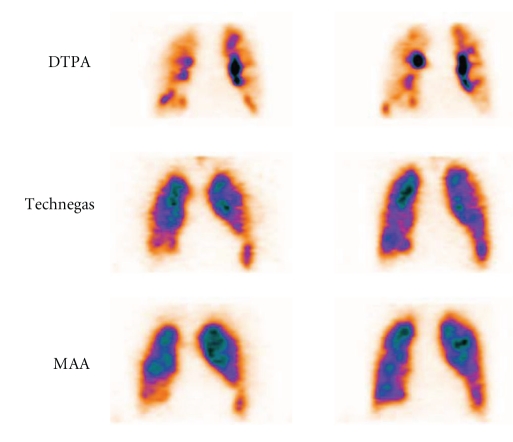
Frontal slices in patient with COPD. Ventilation study with DTPA and technegas with corresponding perfusion images.

Recently, a head to head study of deposition patterns using Technegas and ^99m^Tc-DTPA performed in a group of patients routinely admitted for V/P_SPECT_ and in a group of patients with known COPD was published [19]. Technegas reduced problems of central airway deposition and peripheral hotspots. Unevenness of radiotracer deposition and degree of central deposition were less with Technegas, particularly in the obstructive patients, Figure 1. In some patients, mismatched perfusion defects were only identified using Technegas because the marked peripheral unevenness of ^99m^Tc-DTPA obscured mismatch and thereafter PE might have been overlooked in COPD patients using ^99m^Tc-DTPA. In a few patients, ^99m^Tc-DTPA yielded images of poor quality. It was concluded that Technegas is the superior radio-aerosol, particularly in patients with obstructive lung disease. Another advantage of using Technegas is that a few breaths are sufficient to achieve an adequate amount of activity in the lungs.

## 3. Agent Used for Imaging of Perfusion

For perfusion scintigraphy, radio-labelled MAA, sized 15–100 *μ*m, is injected intravenously. This causes microembolization of pulmonary capillaries and precapillary arterioles, reflecting regional perfusion if at least 60 000 particles are injected [20]. Routinely, about 400 000 particles are injected. As there are over 280 billion pulmonary capillaries and 300 million precapillary arterioles, only a small fraction of the pulmonary bed will be obstructed. Fewer particles might be used in patients with known pulmonary hypertension or after single lung transplantation.

## 4. How to Perform V/P_SPECT_

### 4.1. Image Acquisition

Using a dual head camera, Palmer et al. developed a fast and efficient clinical method for V/P_SPECT_ [21]. The total acquisition time is only 20 minutes. A new algorithm allows calculation of the quotient between ventilation and perfusion and presentation of V/P_quotient_ images for easier diagnosis and quantification of PE extension. 

The ventilation study starts with inhalation of 25–30 MBq ^99m^Tc-DTPA or Technegas. Immediately after ventilation SPECT, a dose of 100–120 MBq ^99m^Tc MAA is given intravenously for perfusion imaging. During the examination, the supine patient carefully maintains the position between ventilation and perfusion acquisitions. Immobilization for only 20 minutes is usually well tolerated by patients. Examination in the supine position is comfortable even for most of critically ill patients. It is also more convenient for the staff. 

When clearance measurements are required, ^99m^Tc-DTPA may be used. Clearance is then calculated from initial and final anteroposterior SPECT projections [21].

### 4.2. Radiation Exposure

The doses of 30 MBq and 120 MBq for ventilation and perfusion, respectively, allow excellent V/P_SPECT_ quality at an effective radiation dose of 1.8 mSv [22].

### 4.3. Reconstruction and Calculation of V/P_quotient_ Images

**Figure 2:**
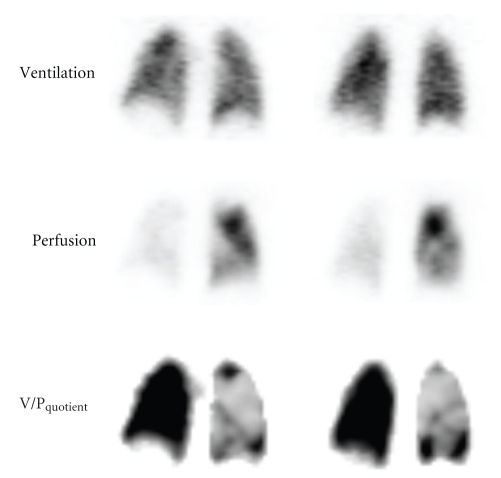
Frontal slices in patient with massive PE. Absent perfusion in the right lung and sub-segmental defects in the left are clearly delineated in V/P_quotient_ images.

Iterative reconstruction is performed using ordered subset expectation maximization (OSEM), for example, with 8 subsets and 2 iterations. In processing the images, the ventilation background was subtracted from the perfusion tomograms and a normalized V/P image set calculated, V/P_quotient_. The algorithms for V/P_quotient_ were developed by Palmer et al. and further amended by Bajc et al. [21, 23]. The main consideration in the creation of  V/P_quotient_ images was to scale smoothened ventilation and perfusion data sets to display V/P_quotient_ in a fixed linear scale allowing separation of normal regions from those with mismatch (Figure 2).

### 4.4. Presentation of V/P_SPECT_

The basic format for V/P_SPECT_ presentation is displayed slices in frontal, sagittal, and transversal projections, available on any modern system. The slices must be accurately aligned so that ventilation and perfusion slices match each other and can be correctly compared. It is of value to achieve this acquisition in one session with maintained body position. This is also a prerequisite for the calculation of V/P_quotient_ images, which greatly facilitates identification and quantification of PE.

Volume rendered images, such as “Maximum Intensity Projection”, are available with almost all SPECT systems, allowing rotating 3D views. Such displays might be useful, particularly for quantification and followup of PE patients [24].

### 4.5. Primary Validation of the V/P_SPECT_ Method

Using a porcine model based upon ^201^Tl-marked emboli as a “gold standard”, Bajc et al. validated the new V/P_SPECT_ method for diagnosis of PE and confirmed the superior value of tomography over planar imaging and improved interobserver agreement of defects on the subsegmental level [25]. In a following clinical head to head comparison between planar imaging and V/P_SPECT_, it was shown that 53% more mismatch points were identified with V/P_SPECT_ compared to the planar technique [26]. Similar results have been found by others [27, 28]. SPECT eliminates superimposed structures, clarifying the segmental and sub-segmental nature of perfusion defect caused by PE.

## 5. Interpretation with Emphasis on PE

**Figure 3:**
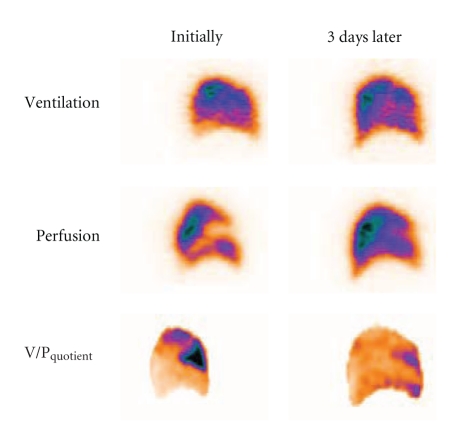
Sagittal slice in patient with segmental PE. Perfusion defect in anterior segment initially, nicely delineated in V/P_quotient_ image. After 3 days, resolution is observed.

Lung scintigraphy for diagnosis of PE and other diseases should routinely include ventilation and perfusion studies [21, 23, 25, 27, 29]. In PE, a perfusion defect is due to an embolus blocking blood flow. Because there is no corresponding blockage in the airway, ventilation remains normal causing a mismatch pattern. The distinction of whether a given perfusion defects is matched or mismatched is fundamental. The next step is to characterize the perfusion defects. Perfusion defects due to blockage of a pulmonary artery should reflect the branching of pulmonary circulation and its classical segmental anatomy. A segmental defect is wedge shaped and with its base on the pleura as will be illustrated (Figure 3).

The European guidelines [2, 3] advocate the new holistic interpretation and reporting of lung SPECT. Freeman et al. argued that “the expert's successful interpretation of lung scans exceeds the best accuracy achievable by algorithms, which, by definition, are distillations of decision making into finite linear steps. The subjective of the whole is superior to any possible attempt to define its discrete parts” [30].

A holistic interpretation of V/P_SPECT_ images includes (1) clinical information and pretest probability for PE, (2) chest X-ray when available, (3) recognition of patterns typical for PE based upon segmental charts, and (4) recognition of patterns of other diseases than PE whenever possible [21, 23].

This is as important as the imaging technique. The clinician can only benefit from reports, which clearly express the presence or absence of PE. This goal was not reached with previous probabilistic reporting methods according to PIOPED or modified PIOPED, which defied how planar scans are reported [1, 31]. Large V/P_SPECT_ studies show that this is achievable if all patterns representing ventilation together with perfusion are considered [23, 32–34]. Conclusive reports were given in 97 to 99% of studies.

Recommended criteria for reading V/P_SPECT_ with respect to acute PE described in the European Guidelines are as follows.

No PE is reported if there are any of the following features:

- normal perfusion pattern conforming to the anatomic boundaries of the lungs,
- matched or reversed mismatch V/P defects of any size, shape, or number in the absence of mismatch,
- mismatch that does not have a lobar, segmental or subsegmental pattern.

PE is reported if there is

- V/P mismatch of at least one segment or two subsegments that conforms to the pulmonary vascular anatomy.

Nondiagnostic for PE is reported if there are

- multiple V/P abnormalities not typical of specific diseases.

In PE, it is fundamental that mismatched areas are conical with the base of the cone along the pleura and conform to known sub-segmental and segmental vascular anatomy. With such interpretation criteria, recent V/P_SPECT_ studies in over 3000 cases showed according to a recent review a negative predictive value of 97–99%, a sensitivity of 96–99%, and a specificity of 91–98% for PE [3]. The rate of nondiagnostic findings was 1–3% [23, 32–34]. Using our technique, V/P_SPECT_ yields ventilation and perfusion images in exactly the same projections. This makes calculation of V/P_quotient_ images possible and facilitates recognition of mismatch, particularly important in the middle lobe and lingula where mismatch may be overlooked if the lung is not accurately delineated by its ventilation images [35]. 

V/P_SPECT_ is the method of choice for quantification of the extent of embolism, because all emboli in the whole lung are recognised and it has greater sensitivity compared to MDCT [27, 32, 33]. The number of segments and sub-segments indicating for PE typical mismatch are counted and expressed in % of the total lung parenchyma [24]. A segmental reduction or a sub-segmental total deficiency of function is attributed 1 point, and a segmental total deficiency is attributed 2 points. Therefore, the 9 segments of each lung can be represented by the total of 18 points. Mismatch defects are expressed as mismatch points, which after division by 36 give the fraction of the lung that is embolized. The reduction in total overall lung function can be estimated by adding the number of regions with reduced ventilation and/or perfusion. 

Patients with up 40% PE could be safely treated at home if ventilation abnormalities engaged not more that 20% of the lung. Since 2004, the University Hospital of Lund has safely treated about 60% of patients with PE at home (approximately 1500).

## 6. Diagnosis of Pulmonary Embolism

V/P_SPECT_ images allow clear identification of segmental and sub-segmental perfusion defects, as in Figure 2 from a woman with extensive PE. 

Figure 3 shows a well-delineated segmental perfusion defect. Followup after three days showed an almost normal pattern, confirming the diagnosis of PE.

Importantly, mismatch findings without a segmental character do not indicate PE. Such findings are often observed in patients with heart failure, mediastinal adenopathy, postradiation therapy, and so forth.

### 6.1. Indications for V/P_SPECT_

#### 6.1.1. Diagnostic Accuracy and Methodological Considerations

The clinical value of V/P_SPECT_ has been confirmed in several studies [27, 29, 32–34]. This has been highlighted by Stein et al. in a recent review [36]. V/P_SPECT_ is today the method recommended by the European Association of Nuclear Medicine for clinical diagnosis, followup, and research [2].

#### 6.1.2. Selection of Therapeutic Strategy

Management of PE was previously confined to in-hospital therapy, using thrombolysis or heparin injections followed by oral anticoagulants for extended periods of time.

V/P_SPECT_ allows objective quantification of PE. It has been shown that out-patient treatment is safe when based upon V/P_SPECT_ that quantifies PE extension and identifies V/P defects of other etiologies [24]. V/P_SPECT_ is accordingly a tool to guide the individual treatment.

#### 6.1.3. Followup

For followup, V/P_SPECT_ is the method recommended by the European Association of Nuclear medicine due to its high sensitivity, noninvasiveness, low radiation exposure, and absence of contraindications [2]. 

Clinical reasons for followup are

- persistent V/P mismatches often occur after PE;
- PE may recur in identical locations;
- a prior study will help determine the age of a new defect;
- There is an impact on therapy decision.

Obviously, evaluation of different drugs and treatment strategies merits the use of V/P_SPECT_ because of its high sensitivity and quality with regards to quantification.

## 7. Additional Findings

V/P_SPECT_ allows diagnosis of several other diseases which have different scintigraphic appearances to PE, as detailed below [2, 3, 37].

### 7.1. Chronic Obstructive Pulmonary Disease (COPD)

**Figure 4:**
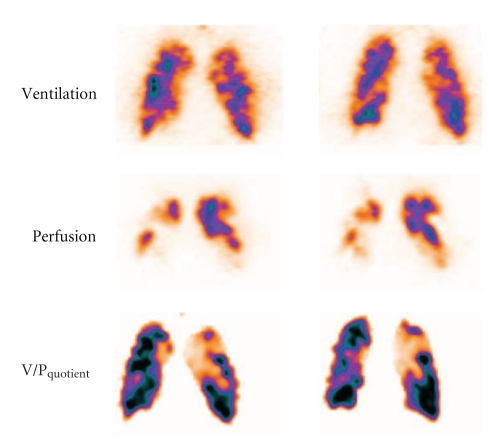
Patient with COPD and extensive PE. On frontal slices, uneven ventilation with peripheral hot spots is observed, and perfusion images showed multiple perfusion defects, clearly delineated on VP_quotient_ images.

In COPD matched areas with defects in ventilation and perfusion are observed. Ventilation defects are commonly more prominent than those of perfusion which leads to a pattern called reverse mismatch [19]. V/P_SPECT_ frequently provides the first indication of COPD. Notably, V/P_SPECT_ allows the diagnosis of PE even in the presence of COPD [32, 37], Figure 4.

### 7.2. Heart Failure

**Figure 5:**
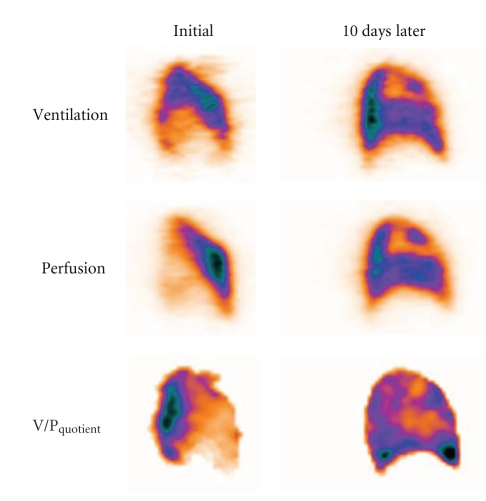
Patient with heart failure. Sagittal slice on the left panel shows redistribution of perfusion towards anterior region; ventilation is less affected causing mismatch of non-segmental character. Right panel shows control after 10 days of treatment with normalization of ventilation and perfusion distribution but with new ventilation and perfusion defect in upper lobe due to pneumonia.

In left heart failure, redistribution of perfusion towards upper lung regions is well recognised since long [38]. Ventilation is usually not affected to the same degree as perfusion, which leads to a mismatch pattern. Importantly, this pattern does not conform to segmental anatomy of pulmonary arteries and it is not of a segmental character. Among patients referred for suspected PE, redistribution of perfusion to upper ventral regions indicated heart failure in 15% of cases [39]. The positive predictive value of the referred V/P_SPECT_ pattern was 88%.  Figure 5 shows V/P_SPECT_ before and after treatment for heart failure.

### 7.3. Pneumonia

**Figure 6:**
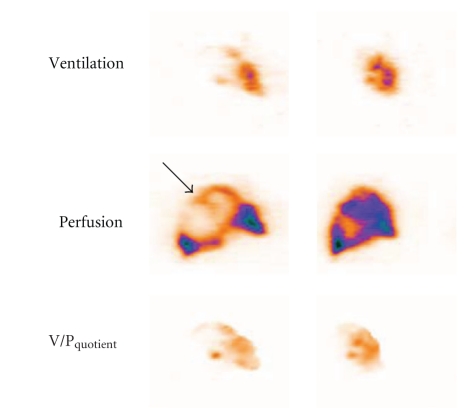
Patient with extensive pneumonia. Sagittal slices in a 79-year-old man who felt general illness, shivering, and low blood pressure. Chest X-ray was interpreted as pleural effusion. The ventilation study of the left lung showed almost absent ventilation, while perfusion was mainly preserved, and a stripe sign is observed. Diagnosis with V/P_SPECT_ was bilateral pneumonia, and this was confirmed later at autopsy.

Pneumonic regions lack ventilation while perfusion may partly be upheld. The most frequent finding is a matched defect [40]. In case of partly preserved perfusion, reversed mismatch is observed [40, 41]. Preserved perfusion along the pleural border leads to a “stripe sign” [42, 43]. V/P_SPECT_ frequently shows this sign because no overlaying structures obscure the images, Figure 6.

The combination of PE and pneumonia is common [32]. Suspicion or knowledge that a patient has pneumonia does not contraindicate V/P_SPECT_. On the contrary, V/P_SPECT_ may be life-saving in the most complex cases [44].

## 8. Concluding Remarks

The qualities of V/P_SPECT_ rely upon adequate and standardized technology of combined ventilation and perfusion studies as well as new holistic interpretation criteria as discussed.

V/P_SPECT_ has excellent sensitivity and specificity. The rate of nondiagnostic reports is ≤3%. V/P_SPECT_ is noninvasive and can be performed in all patients. The radiation exposure is low. With efficient technique and effective organization, V/P_SPECT_ acquisition time is only 20 minutes. Furthermore, it allows quantification of PE that in some centres has impact on choice of treatment. V/P_SPECT_ is uniquely useful for followup and research. Its outstanding qualities merit consideration of its use as the primary diagnostic method for PE in all hospitals in which nuclear medicine is practiced. V/P_SPECT_ frequently gives diagnosis of both PE as well as comorbid conditions as COPD, left heart failure, and pneumonia.
